# A DUSP6 inhibitor suppresses inflammatory cardiac remodeling and improves heart function after myocardial infarction

**DOI:** 10.1242/dmm.049662

**Published:** 2022-12-07

**Authors:** Zongwang Zhang, Yang Chen, Lixia Zheng, Jianyong Du, Shicheng Wei, Xiaojun Zhu, Jing-Wei Xiong

**Affiliations:** ^1^ Beijing Key Laboratory of Cardiometabolic Molecular Medicine, Institute of Molecular Medicine, College of Future Technology, and State Key Laboratory of Natural and Biomimetic Drugs, Peking University, Beijing 100871, China; ^2^ Peking University-Nanjing Institute of Translational Medicine, Nanjing 211800, China; ^3^ Laboratory of Biomaterials and Regenerative Medicine, Academy for Advanced Interdisciplinary Studies, Peking University, Beijing 100871, China

**Keywords:** Myocardial infarction, DUSP6, BCI, Macrophages, Inflammation, PLGA

## Abstract

Acute myocardial infarction (MI) results in loss of cardiomyocytes and abnormal cardiac remodeling with severe inflammation and fibrosis. However, how cardiac repair can be achieved by timely resolution of inflammation and cardiac fibrosis remains incompletely understood. Our previous findings have shown that dual-specificity phosphatase 6 (DUSP6) is a regeneration repressor from zebrafish to rats. In this study, we found that intravenous administration of the DUSP6 inhibitor (*E*)-2-benzylidene-3-(cyclohexylamino)-2,3-dihydro-1*H*-inden-1-one (BCI) improved heart function and reduced cardiac fibrosis in MI rats. Mechanistic analysis revealed that BCI attenuated macrophage inflammation through NF-κB and p38 signaling, independent of DUSP6 inhibition, leading to the downregulation of various cytokines and chemokines. In addition, BCI suppressed differentiation-related signaling pathways and decreased bone-marrow cell differentiation into macrophages through inhibiting DUSP6. Furthermore, intramyocardial injection of poly (D, L-lactic-co-glycolic acid)-loaded BCI after MI had a notable effect on cardiac repair. In summary, BCI improves heart function and reduces abnormal cardiac remodeling by inhibiting macrophage formation and inflammation post-MI, thus providing a promising pro-drug candidate for the treatment of MI and related heart diseases.

This article has an associated First Person interview with the first author of the paper.

## Teaser

**Summary:** Treatment with the small-molecule DUSP6 inhibitor BCI improves heart function and prevents abnormal cardiac remodeling by suppressing macrophage formation and inflammation after myocardial infarction in rats.

## INTRODUCTION

Although the death rate from cardiovascular diseases has declined over the past decades, myocardial infarction (MI) remains the leading cause of mortality and morbidity worldwide (Anderson and Morrow, 2017; Benjamin et al., 2017; Huang et al., 2019). Injury-induced innate immunity plays an essential role in molecular pathogenesis after MI (Huang and Frangogiannis, 2018; King et al., 2017; Nahrendorf et al., 2007; Zhang et al., 2021). Therefore, new strategies targeting macrophages or neutrophils have been actively pursued. Phagocytes within the heart, particularly macrophages, recognize damage-associated molecular patterns (DAMPs) released by dying cardiomyocytes (CMs), triggering pathological inflammatory responses (King et al., 2017). Although clinical trials with broad immunosuppression fail to show any effects on MI, specific targeting of pro-inflammatory cytokines does confer clinical benefits (Huang and Frangogiannis, 2018).

Macrophages, the key regulatory cells in the immune system, display diverse plasticity and physiology. They sequentially differentiate and are mobilized from the bone marrow and spleen to the infarcted myocardium (Nahrendorf et al., 2007; Swirski et al., 2009), where macrophage colony-stimulating factor (M-CSF) activates the mitogen-activated protein kinase (MAPK) pathway, including ERK, p38 and JNK (Smith et al., 2000). During the early injury phase, inflammatory macrophages clear necrotic cellular debris and damaged extracellular matrix from the tissue and attract other immune cells by secreting pro-inflammatory cytokines such as interleukin (IL)-1β, IL-6 and IL-12, and chemokines such as monocyte chemotactic protein 1 (MCP-1; also known as CCL2), C-C motif chemokine 4 (CCL4) and chemokine C-C-motif receptor 2 (CCR2), which further fuel inflammation (Frangogiannis, 2014; Ruparelia et al., 2017; Swirski and Nahrendorf, 2018). These inflammatory mediators play an important role in cardiac remodeling after MI. Therefore, inhibiting pro-inflammatory macrophage functions is critical for the development of new therapies for MI injury.

Dual-specificity phosphatase 6 (DUSP6; also known as MKP3) is a member of the MAPK phosphatase family that acts as a negative feedback regulator of the MAPK cascade (Arkell et al., 2008; Eblaghie et al., 2003). DUSP6 functions as an oncogene or tumor suppressor, depending on the type of cancer (Ahmad et al., 2018; Low and Zhang, 2016), and it also plays a vital role in embryogenesis (Li et al., 2007), as well as heart development and regeneration (Han et al., 2014; Maillet et al., 2008; Zhang et al., 2021). In addition, DUSP6 has been reported to work as an important mediator of inflammatory processes in T-cell immunity and differentiation (Hsu et al., 2018; Li et al., 2015), neutrophils (Zhou et al., 2022) and macrophage-mediated inflammation (Cheng et al., 2018). (*E*)-2-benzylidene-3-(cyclohexylamino)-2,3-dihydro-1*H*-inden-1-one (BCI), a small-molecule inhibitor of DUSP6 that was first isolated through chemical screening in zebrafish, upregulates fibroblast growth factor-targeted gene expression (Molina et al., 2009). BCI treatment also suppresses gastric cancer growth and metastasis (Wu et al., 2018), improves zebrafish cardiac regeneration (Han et al., 2014; Missinato et al., 2018), inhibits lipopolysaccharide (LPS)-induced pro-inflammatory responses in macrophages (Carson et al., 2017; Zhang et al., 2019a) and suppresses osteoclast formation (Cai et al., 2021). However, whether BCI has a therapeutic effect on MI and related diseases remains unclear.

The micro/nanoparticle-based drug delivery system has become a powerful tool to deliver anti-inflammatory drugs to injured tissues (Che et al., 2020). Micro/nanoparticles made from biodegradable poly (D, L-lactic-co-glycolic acid) (PLGA) have been explored as delivery vehicles for therapeutics. Owing to their excellent biocompatibility and controllable biodegradability, PLGA micro/nanoparticles protect macromolecules from instant degradation while allowing tunable releasing rates and profiles *in vivo* (Ding and Zhu, 2018; Mir et al., 2017). In this study, we examined the effects of BCI, via intravenous global or local PLGA-mediated delivery, on the treatment of MI and explored its underlying mechanisms. This work provides evidence that DUSP6/BCI is a promising therapeutic target/drug candidate for MI and related inflammatory diseases.

## RESULTS

### BCI improves cardiac function and attenuates cardiac fibrosis in MI rats

**Fig. 1.**
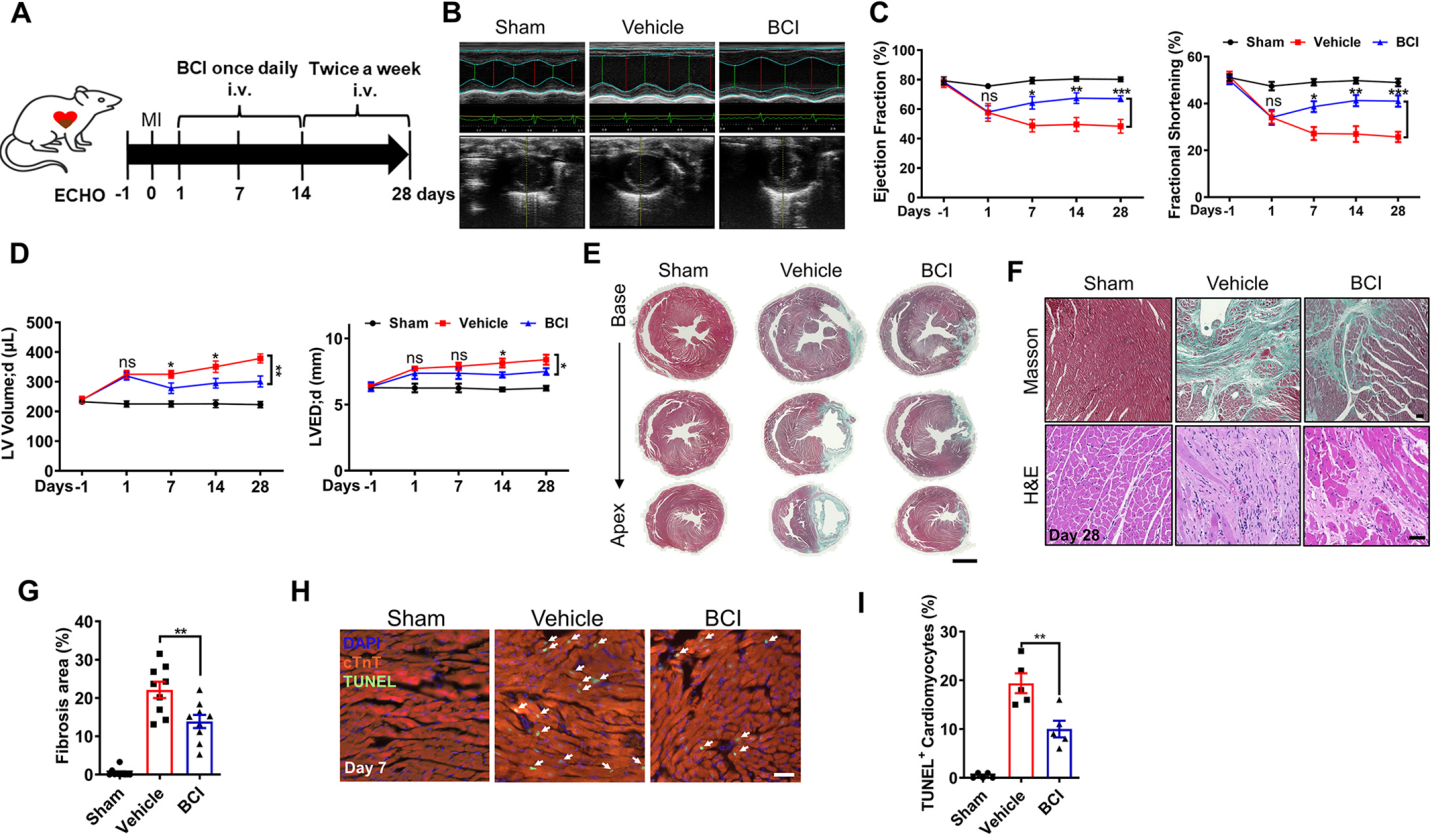
**BCI ameliorates cardiac injury in post-MI rats.** (A) Experimental scheme showing the timing of (*E*)-2-benzylidene-3-(cyclohexylamino)-2,3-dihydro-1*H*-inden-1-one (BCI) injections, myocardial infarction (MI) and echocardiography (ECHO) of rats. i.v., intravenous. (B) Representative M-mode tracings from ECHO of sham-operated and vehicle- or BCI-treated groups at 4 weeks post-MI. (C) The BCI treatment group showed improved ejection fraction and fractional shortening compared with the vehicle treatment group, and partially rescued heart function compared with the sham group (*n*=9–10). (D) BCI treatment improved left ventricle (LV) volumes and LV end-diastolic diameter (LVEDD) compared with vehicle treatment (*n*=9–10). (E) Masson's trichrome staining showing less fibrosis in BCI-treated hearts than in control hearts at 28 days post-MI (scale bar: 3 mm, *n*=9–10). (F) High-magnification images of Masson's trichrome and H&E staining showing decreased fibrosis in BCI-treated hearts at 28 days post-MI (scale bar: 300 μm, *n*=9–10). (G) Quantification of the Masson's trichrome-stained fibrotic area in sham-operated, vehicle-treated and BCI-treated hearts at 28 days post-MI (*n*=9–10 per group). (H) Immunostaining showing TUNEL^+^ cardiomyocytes (CMs) in the infarcted zone in sham-operated, vehicle-treated and BCI-treated heart sections at 7 days post-MI. DAPI was used to stain nuclei, and cTnT was used to stain CMs. Scale bar: 100 μm. (I) Percentage of TUNEL^+^/cTnT^+^ CMs in each group (*n*=5 per group). One-way ANOVA followed by Dunnett's multiple comparison test; mean±s.e.m.; *P<0.05, **P<0.01, ***P<0.001; ns, not significant.

We previously showed in zebrafish that injury-induced H_2_O_2_ promotes heart regeneration via the degradation of DUSP6, which can be mimicked by BCI treatment (Han et al., 2014). We thus reasoned that BCI might have a similar effect on cardiac regeneration and repair in rats post-MI. To test this hypothesis, we applied rat left anterior descending (LAD) coronary artery ligation to establish MI, delivered BCI by intravenous injection, and assessed cardiac function by echocardiography (ECHO) and cardiac fibrosis by Masson's staining (Fig. 1A). The ECHO data revealed that the BCI treatment improved cardiac function, including ejection fraction (EF) and fractional shortening (FS), compared with the vehicle group (Fig. 1B,C; Fig. S1A). Importantly, all three groups had similar EF and FS 1 day before MI (day −1); both the vehicle and the BCI groups had comparable but decreased EF and FS compared with that in the sham group 1[Supplementary-material sup1]day post-MI (day 1); in the BCI group, EF and FS gradually increased, whereas in the vehicle group they decreased from day 1 to day 28 post-MI; and the left ventricle (LV) volume and LV end-diastolic diameter (LVEDD) recovered significantly after BCI administration (Fig. 1D). Masson's staining showed less cardiac fibrosis in the BCI group than in the vehicle group at day 28 post-MI (Fig. 1E–G; Fig. S1B). Consistent with this, Hematoxylin and Eosin (H&E) staining, together with Masson's staining, showed decreased collagen deposition and immune cell infiltration in the BCI group compared with the vehicle group. These data suggested that BCI has a therapeutic effect on MI to improve cardiac function and reduce fibrosis.

### BCI treatment cannot directly reverse ischemic-induced CM death

**Fig. 2.**
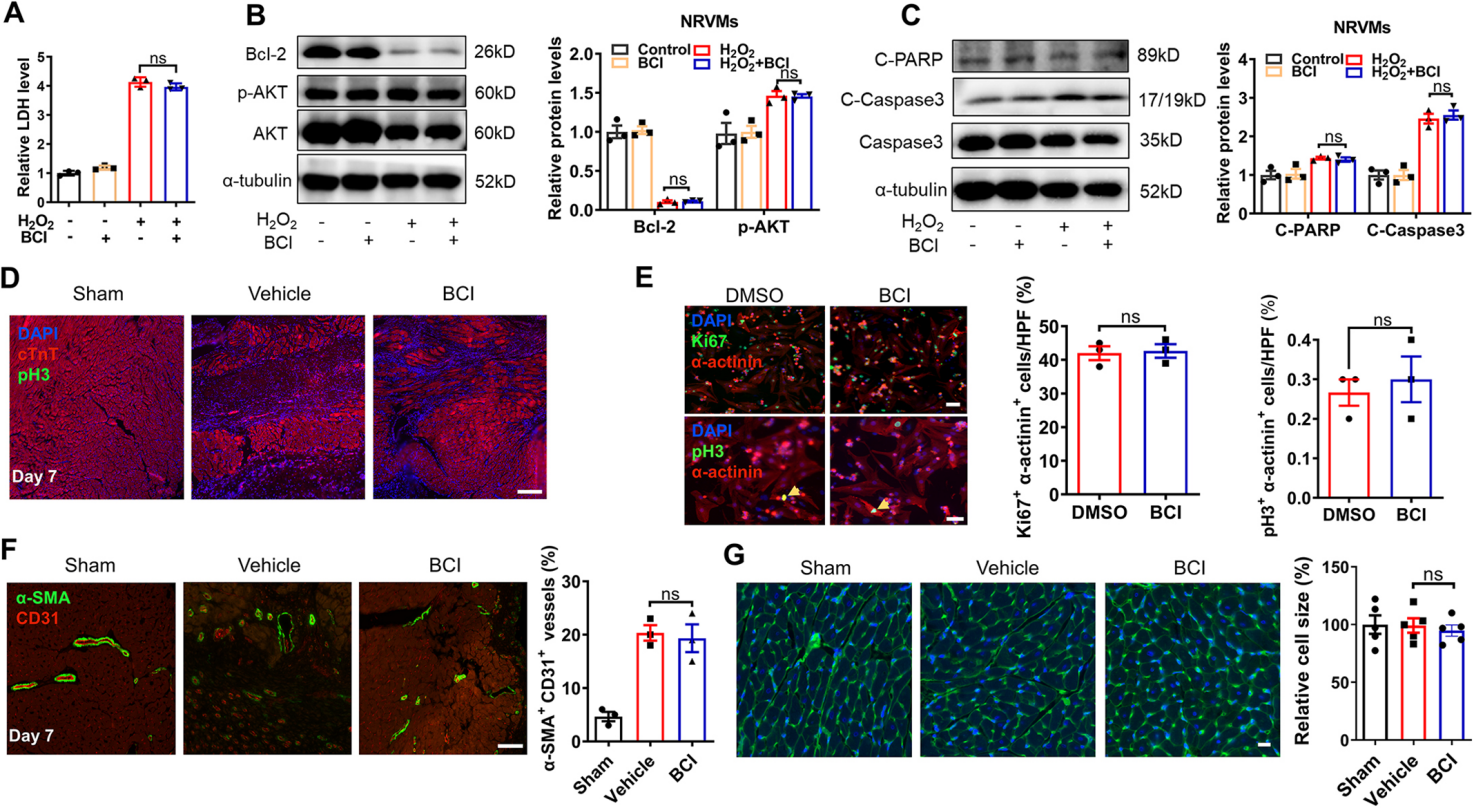
**BCI has no effect on CM proliferation, H_2_O_2_-induced CM death, CM hypertrophy and coronary vessel regeneration.** (A) Released lactate dehydrogenase (LDH) was comparable in neonatal rat ventricular myocytes (NRVMs) treated with DMSO or BCI for 24 h in the presence or absence of H_2_O_2_ (*n*=3 per group). (B) Western blots and quantification of Bcl-2 (α-tubulin was used to normalize protein) and p-AKT (AKT was used to normalize protein) in NRVMs treated with DMSO or BCI for 24 h (*n*=3 per group). (C) Western blots and quantification of H_2_O_2_-induced cleaved PARP (α-tubulin was used to normalize protein) and cleaved caspase 3 (total caspase 3 was used to normalize protein) in NRVMs treated with DMSO or BCI (*n*=3 per group). (D) Immunofluorescent staining showing cTnT^+^ and pH3^+^ CMs of sham-operated, vehicle-treated or BCI-treated LV tissues at 7 days after MI (scale bar: 100 μm). (E) Immunofluorescent staining showed that the numbers of either α-actinin^+^ Ki67^+^ or α-actinin^+^ pH3^+^ CMs were comparable in DMSO- and BCI-treated NRVMs (scale bars: 50 μm; *n*=3 per group). HPF, high-power field. (F) Immunofluorescent staining and statistics for CD31^+^ and α-SMA^+^ vessels of sham-operated, vehicle-treated or BCI-treated LV tissues at 7 days after MI (scale bar: 100 μm; *n*=3 per group). (G) Wheat germ agglutinin staining and statistics for CM size in sham-operated, vehicle-treated or BCI-treated LV tissues at 28 days after MI (scale bar: 20 μm; *n*=5 per group). Mean±s.e.m.; ns, not significant.

We then asked whether BCI treatment has an effect on CM death, proliferation and coronary vessel regeneration after MI. Terminal deoxynucleotidyl transferase dUTP nick end labeling (TUNEL) staining showed that TUNEL^+^/cardiac-specific troponin T (cTnT^+^; also known as TNNT2^+^) CMs were comparable between control and BCI groups at 12 h and 24 h post-MI and BCI administration, but decreased significantly in the BCI group at 3 days post-MI (Fig. S2A); and BCI treatment decreased TUNEL^+^/cTnT^+^ CMs compared with the vehicle control group at 7 days post-MI (Fig. 1H,I). Because *in vivo* experiments could not verify the direct protection of BCI on CMs, we induced CM apoptosis by using H_2_O_2_ *in vitro*. However, BCI treatment did not affect H_2_O_2_-induced apoptosis [apoptotic CM-released lactate dehydrogenase (LDH)] and related signaling pathways such as B-cell lymphoma-2 (Bcl-2), serine/threonine kinase (AKT), caspase 3 and poly ADP-ribose polymerase (PARP) in cultured neonatal rat ventricular myocytes (NRVMs) (Fig. 2A–C), suggesting that BCI does not directly protect CMs from apoptosis. Furthermore, we examined whether BCI improves cardiac function via CM proliferation after MI, and found that BCI treatment did not increase phosphorylated histone 3 (pH3)^+^/cTnT^+^ CMs at 7 days post-MI (Fig. 2D). Similarly, BCI treatment did not promote CM proliferation in cultured NRVMs (Fig. 2E). It is well known that compensatory collateral angiogenesis in the infarct area also contributes to cardiac repair post-MI (Ubil et al., 2014). We examined the density of coronary arteries by using anti-cluster of differentiation 31 (CD31; also known as PECAM1; an endothelial cell marker) and α-smooth muscle actin (α-SMA; also known as ACTA2; a smooth muscle cell marker), and found that BCI treatment caused no changes in arterial angiogenesis post-MI (Fig. 2F). Furthermore, wheat germ agglutinin (WGA) staining showed that BCI administration for 4 weeks had no effect on CM hypertrophy (Fig. 2G). Together, our data suggest that BCI treatment does not directly affect CM death/proliferation and arterial angiogenesis.

### BCI reduces inflammatory cytokine production and macrophage and neutrophil infiltration post-MI

**Fig. 3.**
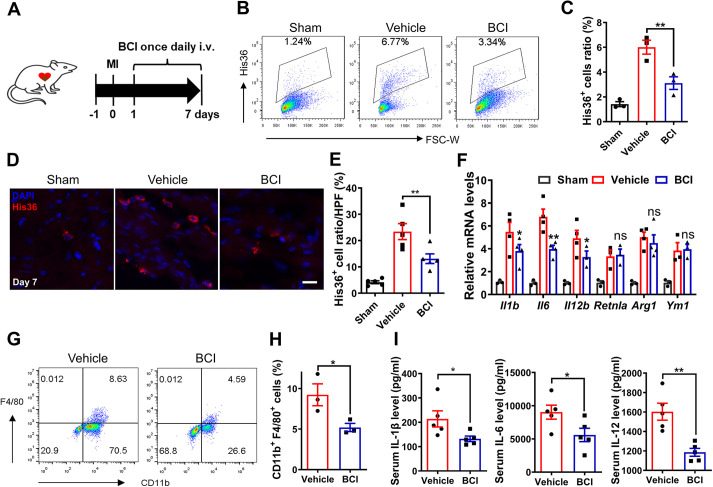
**BCI inhibits macrophage recruitment in infarcted rat hearts and LPS-induced mouse abdominal macrophages.** (A) Experimental scheme showing the timing of BCI injections and MI in rats. (B,C) Percentages of His36^+^ macrophages in the LV of rats at 7 days post-MI by flow cytometry (B) and statistics of His36^+^ macrophages (C) (*n*=3 per group; two hearts per sample). (D) Immunofluorescent staining showing decreased numbers of His36^+^ macrophages (red) in the LV of rats after BCI treatment at 7 days post-MI compared with vehicle treatment (scale bar: 50 μm). (E) The percentage of His36^+^ macrophages to DAPI^+^ cells in each group (*n*=5 per group). (F) The expression levels of *Il1b*, *Il6*, *Il12b*, *Retnla*, *Arg1* and *Ym1* mRNAs were determined in sham and MI hearts (day 7) with or without BCI treatment (*n*=3–4 per group). (G,H) Percentages of lipopolysaccharide (LPS)-induced CD11b^+^ (also known as ITGAM^+^) F4/80^+^ macrophages in mouse abdominal cells were measured by flow cytometry (G), and statistics of CD11b^+^ F4/80^+^ cells are shown (H) (*n*=3 per group; two mice per sample). (I) LPS-induced IL-1β, IL-6 and IL-12 proteins in mouse serum detected by ELISA (*n*=5 mice per group). One-way ANOVA followed by Dunnett's multiple comparison test; mean±s.e.m.; *P<0.05, **P<0.01; ns, not significant.

Macrophage infiltration is essential for initiating cardiac repair after MI. Our previous data showed that rat DUSP6 is highly enriched in neutrophils and macrophages, whereas it is hardly detectable in CMs after MI (Zhou et al., 2022). After intravenous delivery of BCI daily for 7 days post-MI (Fig. 3A), we dissected the LV, subjected it to digestion into single cells and applied fluorescence-activated cell sorting analysis. The percentage of macrophages in infarcted hearts in the BCI treatment group was significantly lower than that in the vehicle group, but it was higher than that in the sham group (Fig. 3B,C). Consistent with this, immunohistochemical analysis showed more infiltrated macrophages in infarcted hearts at 7 days post-MI, and the number decreased significantly after BCI treatment (Fig. 3D,E). This was not due to BCI-induced macrophage apoptosis/necrosis because His36^+^ and TUNEL^+^ macrophages were comparable between control and BCI groups (Fig. S3A). Quantitative real-time PCR (qRT-PCR) from rat hearts showed that BCI treatment inhibited the mRNA expression of pro-inflammatory cytokines of M1-type macrophages, such as *Il1b*, *Il6* and *Il12b*, but had no significant effect on the M2-type macrophage markers resistin-like alpha (*Retnla*), arginase-1 (*Arg1*) and chitinase 3-like 3 (*Ym1*; also known as *Chil3*) (Fig. 3F). To further investigate the effects of BCI on macrophage infiltration and cytokine secretion, we turned to an acute inflammatory mouse model induced by LPS and found that BCI treatment decreased the number of infiltrated macrophages in the peritoneal fluid after LPS induction for 12 h (Fig. 3G,H). Accordingly, BCI treatment inhibited the expression of the inflammatory cytokines IL-1β, IL-6 and IL-12 in mouse serum (Fig. 3I). Together, these data suggest that BCI treatment attenuates macrophage cytokine secretion and infiltration in both acute mouse and rat models of inflammation.

We then asked whether BCI treatment affects neutrophil recruitment post-MI. We found that BCI treatment decreased the percentage of neutrophils in infarcted hearts compared with vehicle-treated hearts (Fig. S4A,B). After LPS stimulation of abdominal neutrophils (ABNs), the mRNA expression levels of pro-inflammatory cytokines *Il1b*, *Il6*, *Il12b* and *Cd14* increased, but they were repressed by BCI treatment (Fig. S4C). We also found that BCI inhibited phosphorylated (p-)p65 (also known as SYT1) in ABNs that had been induced by LPS stimulation (Fig. S4D). In an acute inflammatory mouse model, we found that BCI treatment decreased the numbers of infiltrated neutrophils in the peritoneal fluid after LPS induction for 12 h (Fig. S4E). Thus, these data suggest that BCI treatment improves cardiac function post-MI at least partly through attenuating macrophage- and neutrophil-mediated inflammation.

### BCI treatment inhibits BMC differentiation into macrophages

**Fig. 4.**
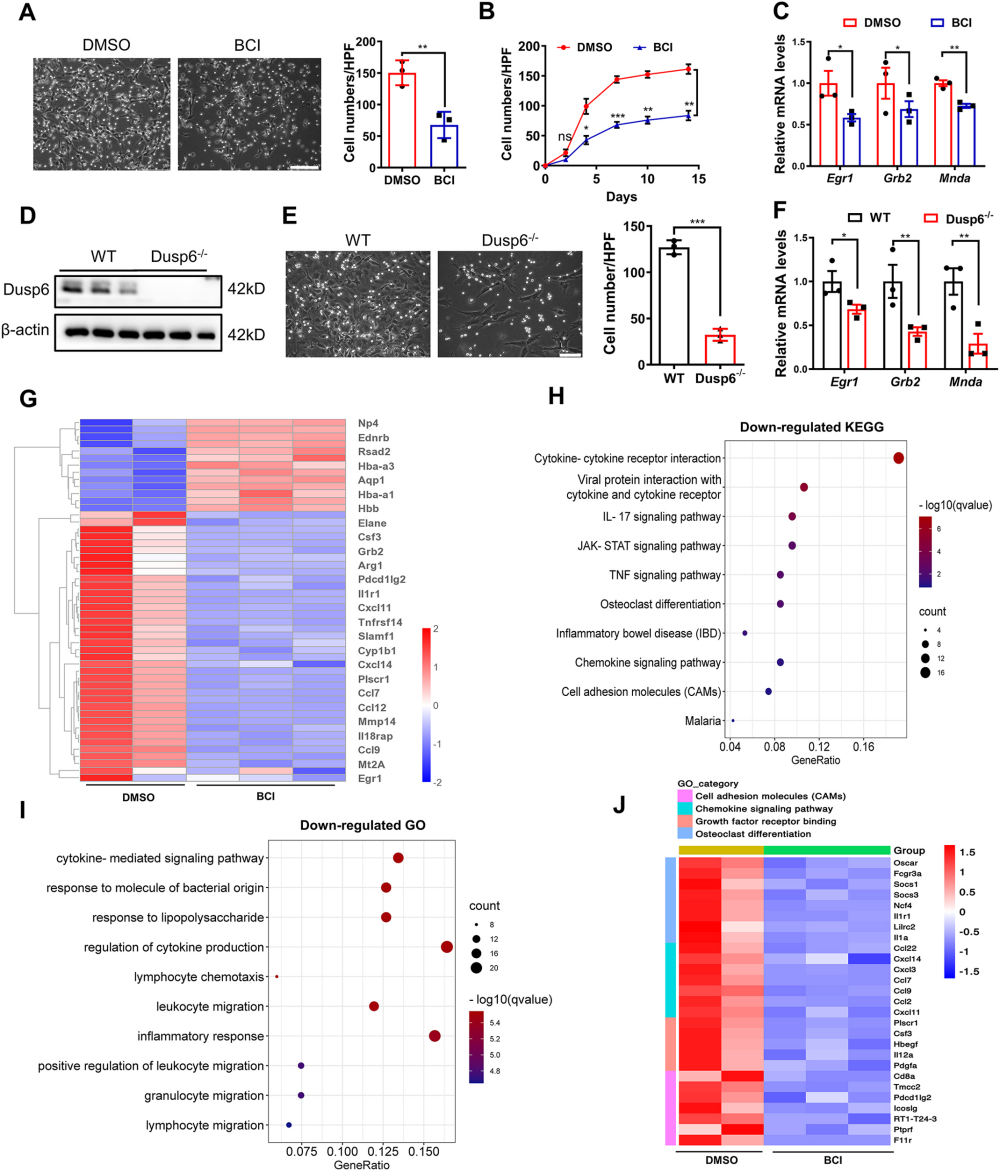
**BCI inhibits differentiation of BMCs into macrophages.** Bone-marrow cells (BMCs) were isolated and cultured from the femurs and tibias of 6-week-old wild-type Sprague-Dawley rats. (A) Bright-field images of BMC-derived macrophages, and the numbers of adherent macrophages in each HPF after 7-day induction by M-CSF in the presence of DMSO or BCI (scale bar: 100 μm; *n*=3 per group). (B) Numbers of adherent macrophages in each HPF at different time points of BCI treatment (*n*=3 per group). (C) Expression levels of *Egr1*, *Grb2* and *Mnda* mRNAs in BMCs at day 3 after M-CSF induction with DMSO or BCI treatment (*n*=3 per group). (D) Western blots showing DUSP6 expression in M-CSF-induced bone-marrow-derived macrophages (BMDMs) from wild-type (WT) or *Dusp6* mutant rats (*n*=3 per group). (E) Bright-field images of BMC-derived macrophages and the numbers of adherent macrophages in each HPF after 7-day induction by M-CSF from WT or *Dusp6* mutant rats (scale bar: 100 μm; *n*=3 per group). (F) Expression levels of *Egr1*, *Grb2* and *Mnda* mRNAs in BMDMs at day 3 after M-CSF induction from WT or *Dusp6* mutant rats (*n*=3 per group). (G) RNA-seq showing differential gene expression in myeloid cells at 3 days after M-CSF induction with or without BCI. (H) Kyoto Encyclopedia of Genes and Genomes (KEGG) analysis of genes downregulated by BCI revealed the top 10 affected pathways. (I) Gene Ontology (GO) analysis of genes downregulated by BCI showed ten signaling pathways including cytokines/chemokines. (J) Heatmaps showing the downregulation of cell adhesion molecules, chemokine signaling pathway, growth factor receptor binding and osteoclast differentiation after BCI treatment. One-way ANOVA followed by Dunnett's multiple comparison test; mean±s.e.m.; *P<0.05, **P<0.01, ***P<0.001.

The MAPKs are the key signaling components downstream from M-CSF1 (also known as CSF1) receptor, and their sustained activation is essential for macrophage formation (Grasset et al., 2010). We isolated bone-marrow cells (BMCs) and differentiated them into bone-marrow-derived macrophages (BMDMs) by M-CSF induction *in vitro*. We found BCI treatment, and also *Dusp6* deletion, reduced the formation of BMDMs during the 2 weeks of culture (Fig. 4A,B,D,E). Consistent with this, BCI treatment or *Dusp6* deletion inhibited the expression levels of macrophage differentiation genes, including early growth response protein 1 (*Egr1*), growth factor receptor-bound protein 2 (*Grb2*) and myeloid nuclear differentiation antigen (*Mnda*) after M-CSF induction for 3 days (Fig. 4C,F). To further assess the effect of BCI on global transcriptomic changes in BMCs under M-CSF induction, we applied bulk RNA sequencing (RNA-seq) and found that BCI treatment led to downregulation of *Egr1* and *Grb2* (Fig. 4G). Bulk RNA-seq analysis revealed that BCI treatment inhibited the signaling pathways associated with cell differentiation, such as cell adhesion molecules, growth factor receptor binding and osteoclast differentiation (Fig. 4H,J). Furthermore, Gene Ontology (GO) analysis revealed that broad cytokine and chemokine genes were inhibited by BCI (Fig. 4I), suggesting that BCI inhibits the differentiation of immature BMCs into mature macrophages. Collectively, these data suggest that BCI acts through DUSP6 to inhibit BMC differentiation into macrophages by downregulating differentiation genes and related signaling pathways.

### BCI inhibits macrophage activation via p38–NF-κB signaling

**Fig. 5.**
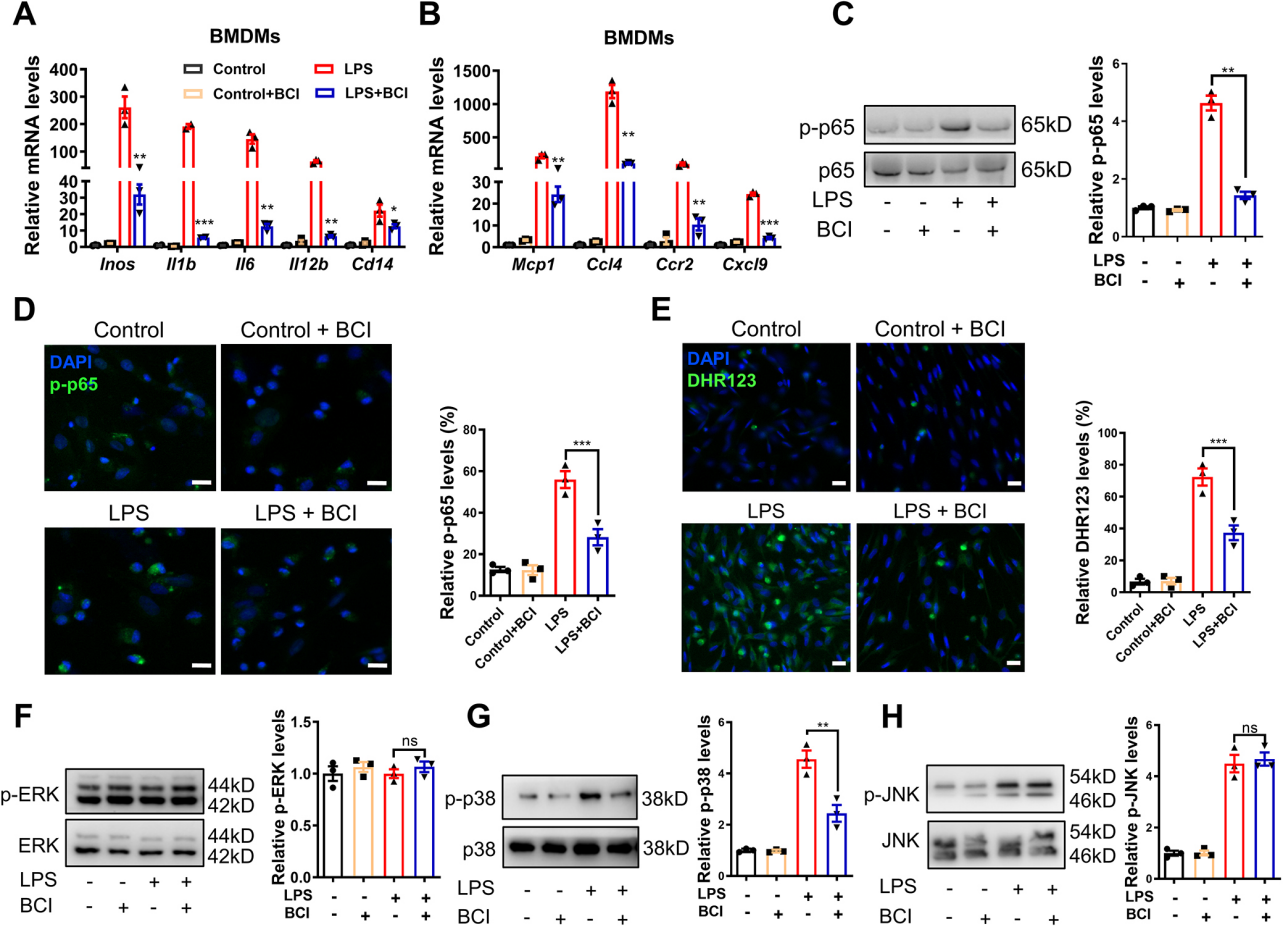
**BCI attenuates inflammation through the p38–NF-κB signaling pathway.** BMDMs were stimulated by LPS in the presence of DMSO or BCI, and the treated cells were used for qRT-PCR, immunostaining and western blotting. (A) qRT-PCR showing expression levels of LPS-induced *Inos*, *Il1b*, *Il6*, *Il12b* and *Cd14* mRNAs in BMDMs with DMSO or BCI (*n*=3 per group). (B) qRT-PCR showing expression levels of LPS-induced *Mcp1*, *Ccl4*, *Ccr2* and *Cxcl9* mRNAs in BMDMs with DMSO or BCI (*n*=3 per group). (C) Western blots and quantification of LPS-induced p-p65 and p65 in BMDMs with DMSO or BCI treatment (*n*=3 per group). (D) Immunofluorescent staining and quantification of LPS-induced p-p65 (green) in BMDMs with DMSO or BCI treatment (*n*=3 per group; scale bars: 20 μm). (E) Immunofluorescent staining and quantification of LPS-induced reactive oxygen species measured by dihydrorhodamine 123 (DHR123) (green) in BMDMs with DMSO or BCI treatment (*n*=3 per group; scale bars: 20 μm). (F-H) Western blots and quantification of LPS-induced p-ERK (F), p-p38 (G) and p-JNK (H) in BMDMs with DMSO or BCI treatment (*n*=3 per group). One-way ANOVA followed by Dunnett's multiple comparison test; mean±s.e.m.; *P<0.05, **P<0.01, ***P<0.001; ns, not significant.

Next, we isolated the rat BMDMs to explore how BCI inhibits macrophage activation. Upon LPS stimulation of BMDMs, the expression levels of pro-inflammatory cytokines (*Il1b*, *Il6*, *I112b* and *Cd14*), chemokines (*Mcp1*, *Ccl4*, *Ccr2* and *Cxcl9*) and inducible NO synthase (*Inos*; also known as *Nos2*) were significantly induced, but they were repressed by BCI treatment (Fig. 5A,B). As expected, BCI inhibited p-p65 and reactive oxygen species (ROS) induced by LPS stimulation in BMDMs (Fig. 5C–E). DUSP6 is a classical p-ERK phosphatase and also affects p-p38 and p-JNK with lower activity (Maillet et al., 2008; Zhou et al., 2022). Western blot analyses revealed that BCI treatment decreased the LPS-induced p-p38 proteins that are known to mediate inflammation, but had no effects on the levels of p-ERK and p-JNK (Fig. 5F–H). In addition, we found that deletion of *Dusp6* had no effect on LPS-induced inflammation because BCI still had anti-inflammatory effects in *Dusp6* mutant BMDMs, in which BCI decreased the expression levels of *Il1b*, *Il6*, *I112b* and *Cd14*, as well as p-p65 (Fig. S5A,B). Taken together, these data suggest that BCI inhibits macrophage activation and cytokine/chemokine secretion independent of DUSP6 by inhibiting p38–NF-κB signaling.

### Biodegradable PLGA facilitates slow-release delivery of BCI for cardiac repair post-MI

**Fig. 6.**
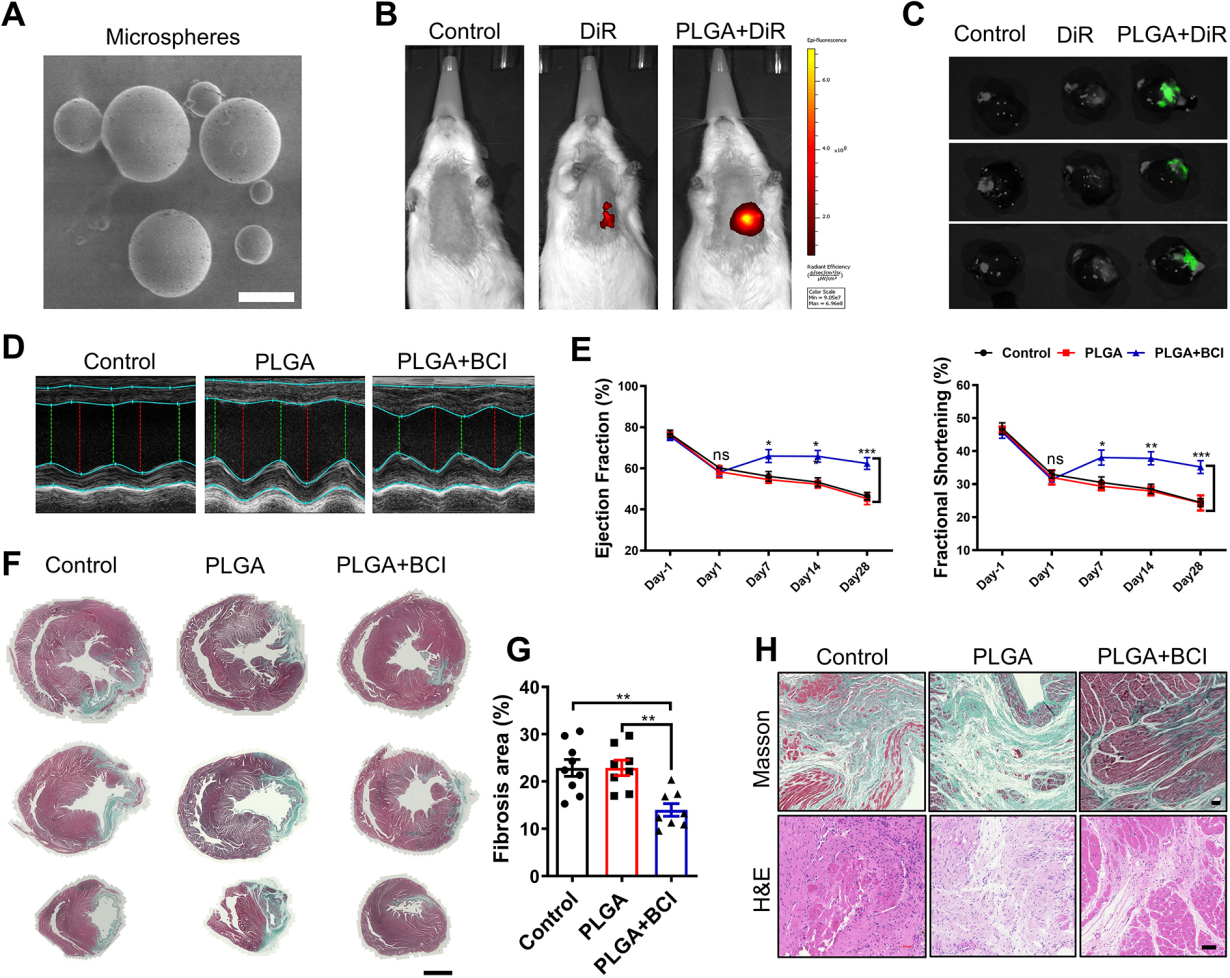
**Intramyocardial injection of PLGA-encapsulated BCI improves cardiac function post-MI in rats.** (A) Poly (D, L-lactic-co-glycolic acid) (PLGA) microspheres under scanning electron microscopy (scale bar: 5 μm). (B) *In vivo* fluorescence imaging of rats 4 weeks after intramyocardial injection of normal saline (control), 1,1'-dioctadecyl-3,3,3′,3'-tetramethylindotricarbocyanine iodide (DiR) or PLGA-encapsulated DiR (*n*=3 per group). (C) Imaging of hearts 4 weeks after intramyocardial injection of normal saline (control), DiR or PLGA-encapsulated DiR (*n*=3 per group). (D) Representative M-mode tracings from ECHO of control, PLGA-treated and PLGA+BCI treated rat hearts at 4 weeks after MI. (E) Statistical analysis of ejection fraction and fractional shortening of control, PLGA-treated and BCI+PLGA-treated rat hearts 1 day before MI, and on days 1, 7, 14 and 28 after MI (*n*=8–9). (F) Masson's trichrome staining of control, PLGA-treated and BCI+PLGA-treated hearts at 28 days post-MI. Scale bar: 3 mm. (G) Quantification of Masson's trichrome-stained fibrotic area in F (*n*=8–9). (H) Masson's trichrome staining and H&E staining of control, PLGA-treated and BCI+PLGA-treated hearts at 28 days post-MI. Scale bars: 300 μm. One-way ANOVA followed by Dunnett's multiple comparison test; mean±s.e.m.; *P<0.05; **P<0.01, ***P<0.001; ns, not significant.

Considering the poor solubility and low bioavailability of BCI, we sought biomaterials to achieve long-term, sustainable, local delivery of BCI into infarcted hearts. PLGA is a good candidate due to its excellent biocompatibility and controllable biodegradability (Ding and Zhu, 2018). We first prepared PLGA-coated BCI or 1,1'-dioctadecyl-3,3,3′,3'-tetramethylindotricarbocyanine iodide (DiR) microspheres (Fig. 6A). By injecting PLGA-coated fluorescent DiR into the rat heart, we found that DiR encapsulated by PLGA was able to persist for 4 weeks by *in vivo* imaging, and the fluorescence intensity was much stronger in the PLGA-coated DiR group than in the DiR group (Fig. 6B,C). Importantly, only a single intracardial injection of PLGA-coated BCI right after MI in rats resulted in improved cardiac function, indicated by elevated EF and FS (Fig. 6D,E), decreased cardiac fibrosis and inhibited infiltrating immune cells (Fig. 6F–H) at 4 weeks post-MI. These results suggest that local administration of PLGA-coated BCI is an effective treatment for MI.

## DISCUSSION

MI causes CM loss and structural remodeling, as well as LV dysfunction and dilation, eventually leading to heart failure (Alonso-Herranz et al., 2020). Previous studies have shown that deletion or inhibition of DUSP6 promotes heart regeneration in zebrafish (Han et al., 2014), mice (Maillet et al., 2008; Missinato et al., 2018) and rats (Zhou et al., 2022). Here, we reported the beneficial effects of DUSP6 allosteric inhibitor BCI, by improving cardiac function and ameliorating cardiac inflammation and fibrosis at least partly through attenuating p38–NF-κB signaling, and local delivery of a single dose of PLGA-encapsulated BCI had notable effects on cardiac repair post-MI. Thus, either intravenous or PLGA-encapsulated BCI delivery has potential for becoming an effective therapy for MI.

Monocytes/macrophages and neutrophils are innate immune cells that first respond to tissue injury by detecting ‘danger’ signals and initiate the inflammatory processes (Shi and Pamer, 2011). DAMP-triggered inflammatory responses aggravate CM death after MI. Interventions in the differentiation, migration and excessive inflammation of leukocytes are favorable strategies for alleviating tissue damage (Nathan and Ding, 2010). Our results suggest that BCI neither directly prevents CM apoptosis nor promotes CM proliferation, and that BCI inhibits cardiac inflammation and thus attenuates CM death after acute MI. BCI treatment decreased BMC differentiation into macrophages by downregulating *Egr1*, *Grb2* and *Mnda*, as well as related cell-adhesion molecules, growth factor receptor binding and osteoclast differentiation signaling. However, BCI treatment decreased cytokine-mediated NF-κB and p38 activation, and the numbers of infiltrating macrophages around the infarcted area independent of DUSP6. Therefore, BCI is another drug candidate for MI via suppressing macrophage formation and activation. Along this line of research, reducing inflammation by targeting immune cells post-MI, using agents such as MCB-613 (Mullany et al., 2020), dimethyl itaconate (Nakkala et al., 2021) and AXL receptor tyrosine kinase inhibitor (DeBerge et al., 2021), has shown beneficial effects by decreasing infarct size and adverse cardiac remodeling.

Bioabsorbable microspheres have recently drawn much attention as drug-delivery materials for the treatment of human diseases (Lee et al., 2013; Zhu et al., 2021). PLGA microparticles are reported to produce large porous particles with a uniform distribution of pores, resulting in higher entrapment efficiency and prolonged drug release (Gupta and Ahsan, 2011; Lee et al., 2015). A single local intracardial injection of PLGA-encapsulated BCI, immediately after MI, had remarkable therapeutic effects, thus avoiding loss of the drug in the circulatory system and the poor solubility of BCI. These findings support the feasibility of developing PLGA microparticles as vehicles for delivering BCI and other small molecules into the infarcted myocardium.

In conclusion, we here demonstrated that BCI is an attractive potential therapy for the treatment of MI, presented novel mechanistic insights into post-MI inflammation, and identified PLGA-based delivery of BCI for controlling inflammation and cardiac remodeling after MI. Thus, BCI can be explored for developing therapeutic drugs for heart disease and other related inflammatory diseases.

## MATERIALS AND METHODS

### Experimental animals

Neonatal (postnatal day 3) Sprague-Dawley (SD) rats, and adult mice and rats used in this work were purchased from Vital River Laboratory Animal Technology Co., Ltd (Beijing, China). *Dusp6* knockout rats were constructed in our laboratory as previously reported (Zhou et al., 2022). All procedures involving experimental animals were performed according to protocols approved by the Institutional Animal Care and Use Committee at Peking University, Beijing, China.

### Adult rat MI

The rat MI model was created by ligation of the LAD coronary artery in male wild-type rats at the age of 8–10 weeks as previously described (Du et al., 2022). Briefly, each rat was anesthetized by intraperitoneal injection of tribromoethanol (300 mg/kg; Sigma-Aldrich, St Louis, MO, USA). After complete anesthesia, each rat was immobilized in the supine position, intubated and connected to a small-animal ventilator on an operating table (MouseVent, Kent Scientific Corp., Torrington, CT, USA). Under controlled ventilation, the thoracotomy was made in the left intercostal space between the third and fourth ribs, and the heart was exposed by removing the pericardium. The LAD coronary artery was permanently ligated using a 6-0 non-absorbable surgical suture, and the chest and skin were immediately sutured. The rat was finally removed from the ventilator and kept warm until revived. Rats with sham surgery were subjected to the same procedure without performing the ligation.

### ECHO

ECHO was applied to anesthetized rats (with 1.0% isoflurane) using a Vevo 3100 system (Visual Sonics, Toronto, ON, Canada) as previously described (Zhang et al., 2016). The hair on the left chest was completely removed using a depilatory paste. A 20-MHz variable-frequency transducer was used to capture 2D ECHO images on both the mid-ventricular short axis and the parasternal long axis. The resulting images were analyzed to derive indexes of cardiac function and LV dilation on the basis of a standard formula. ECHO data were collected and analyzed on day −1 (baseline at 1 day before MI) as well as on days 1, 7, 14 and 28 post-MI.

### BCI treatment in rats post-MI

BCI was from MedChemExpress Co. (MCE; Shanghai, China). To assess BCI efficacy, male SD rats were randomly divided into three groups: sham-operated, vehicle-treated and BCI-treated. BCI was administered intravenously once daily for 2 weeks and twice weekly in the following 2 weeks. According to the dose of BCI administered in different animal models, we chose to inject 8 mg/kg intravenously (Cai et al., 2021; Duan et al., 2021; Ramkissoon et al., 2019). BCI was dissolved in dimethyl sulfoxide (DMSO), solutol (MCE) and 0.9% normal saline in the volume ratio 1:1:3. The vehicle group was administered solvent control. Experimental rats were euthanized to collect heart samples for analysis.

Regarding usage of the slow-release material PLGA, male SD rats were randomly divided into three groups: control, PLGA and PLGA+BCI. PLGA or PLGA+BCI (BCI, 8 mg/kg) was injected at the infarct site during the MI surgery. The rats were euthanized, and their hearts were collected for analysis.

### BCI treatment on mice after LPS induction

Male C57BL/6J mice at the age of 8 weeks were intraperitoneally injected with LPS (1 mg/kg, Sigma-Aldrich), and solvent or BCI was then injected (8 mg/kg). The treated mice were sacrificed for serum collection 12 h later. Then, 2 ml phosphate-buffered saline (PBS) with or without BCI was injected into the abdominal cavity of mice, and the abdominal fluid was collected and centrifuged for analysis of macrophages.

### Preparation of heart cell suspension

On day 7 after MI, the rat heart was removed, and the LV below the ligation site was collected for isolating heart cells. Briefly, the infarcted tissue was dissected and washed with Hanks' balanced salt solution (HBSS) without Ca^2+^ and Mg^2+^ (MacGene, Beijing, China). Using micro-dissecting scissors, the LV tissue was minced to obtain ∼1 mm^3^ pieces and was then treated with 5 ml digestion solution containing collagenase II (0.3 mg/ml; Thermo Fisher Scientific, Pittsburgh, PA, USA) and trypsin (1 mg/ml; Amresco, Pennsylvania, PA, USA) in HBSS for 5 min at 37°C. The resulting supernatant was collected, and the residual tissues were repeatedly treated with the digestion solution until little remained. The cell supernatant was passed through a 100-μm Falcon cell strainer (BD Biosciences, San Jose, CA, USA) to obtain single-cell suspensions. After centrifugation at 240 ***g*** for 5 min, the cell pellets were collected and resuspended in RIPA 1640 medium (Gibco, Grand Island, NY, USA). The heart cells were passed through a 40-μm Falcon cell strainer. The harvested cells were washed with ice-cold PBS and used for flow cytometric analysis.

### Pathological evaluation of rat hearts

Rat hearts were collected and fixed in 4% paraformaldehyde (PFA) for 2 days. All heart tissues were dehydrated, cleared and infiltrated in a Histoprocessor (Tissue-Tek, Sakura, Tokyo, Japan). Paraffin-embedded tissues were cut at 5 µm, and the serial sections were stained with H&E. Masson's trichrome staining was applied as previously described (Chuang et al., 2016). Briefly, heart sections were immersed in Bouin's solution and then stained with Mayer's Hematoxylin solution, Biebrich Scarlet–acid fuchsin, phosphomolybdic acid–phosphotungstic acid and Aniline Blue reagents (Sigma-Aldrich) sequentially, with a distilled H_2_O wash between each reagent. The sections were dried, mounted on glass slides, examined and photographed under a microscope (IX73, Olympus Tokyo, Japan).

### Isolation of NRVMs

NRVMs were isolated from postnatal day 3 SD rat hearts according to a previously described method (Du et al., 2022). Briefly, the hearts of SD rat pups were dissected and washed with Ca^2+^- and Mg^2+^-free HBSS. Using micro-dissecting scissors, ventricular tissue was minced into small pieces, and then treated with 5 ml digestion solution containing collagenase II (0.3 mg/ml) and trypsin (1 mg/ml) in HBSS for 5 min at 37°C. The resulting cell supernatant was collected, and the residual tissue was repeatedly treated with the digestion solution until little remained. The supernatants were transferred to a tube with an equal volume of ice-cold Dulbecco's modified Eagle medium (DMEM; Gibco) containing 10% fetal bovine serum (FBS; MacGene) and 1% penicillin–streptomycin, and then centrifuged at 240 ***g*** for 5 min at room temperature. The cell pellets were resuspended in 25 ml DMEM containing 5% FBS, 1% penicillin–streptomycin and 1 μmol/l cytosine arabinoside. The cells were incubated in a 100-mm dish for 1.5 h at 37°C to eliminate fibroblast contamination, then non-adherent cells were collected and seeded at a final concentration of 5×10^5^ cells/ml. After incubation for 72 h, the medium was removed, and the NRVMs were cultured with DMEM containing 5% FBS and 1% penicillin–streptomycin for further analysis.

### Preparation and culture of rat BMDMs

Rat BMDMs were generated from bone marrow by M-CSF induction. Bone marrow was aseptically flushed from the tibiae and femurs of euthanized rats and depleted of red blood cells using Red Blood Cell Lysis Buffer (Beyotime, Shanghai, China). Cells were resuspended and cultured in RIPA 1640 supplemented with 10% FBS, 1% penicillin–streptomycin and 20 ng/ml M-CSF (PeproTech, Rocky Hill, NJ, USA) for 7 days. Nonadherent cells were removed, and the M-CSF-conditioned medium was changed on day 3. Adherent cells were seeded in six-well plates at 1×10^6^ cells per well, and macrophages were stimulated with 1 μg/ml LPS for 24 h before mRNA analysis or protein analysis.

### Isolation of rat abdominal neutrophils

Glycogen-elicited rat abdominal neutrophils were prepared. First, 10 ml of 1% glycogen (dissolved in 0.9% NaCl) was injected intraperitoneally to accumulate neutrophils for 6 h. The rats were then anesthetized and euthanized. Peritoneal cells were harvested by intraperitoneal lavage using RPMI 1640 medium (10 ml per rat). The peritoneal exudate was filtered through gauze and centrifuged at 500 ***g*** for 10 min. After lysis of contaminating erythrocytes with Red Blood Cell Lysis Buffer, the neutrophils were collected by centrifugation and washed twice with PBS. Cells were seeded in six-well plates at 1×10^6^ cells per well, and neutrophils were stimulated with 1 μg/ml LPS for 6 h before cells were collected for mRNA analysis or protein analysis.

### CCK-8 cytotoxicity assays

We dispensed 100 μl cell suspension (2×10^4^ cells per well) into a 96-well plate and pre-incubated the plate for 24 h at 37°C in a 5% CO_2_ atmosphere. We added 100 μl of various concentrations of toxicant into the culture medium and incubated the plate for 24 h. Then, 20 μl cell counting kit-8 (CCK-8) solution (Sigma-Aldrich) was added to each well of the plate, incubated for 1–4 h in an incubator, and the absorbance was measured at 450 nm using a microplate reader. CCK-8 data and immunostaining showed that the BCI concentration (∼2.5 μmol/l for NRVMs; ∼1 μmol/l for BMDMs) was nontoxic, and it was thus used in subsequent experiments (Fig. S6A–D).

### qRT-PCR

Rat mRNA was isolated from cells or tissues with a Total RNA kit (Tiangen, Shanghai, China) and reverse transcribed with an RT Master Mix kit (Yeasen, Shanghai, China). qRT-PCR was performed with an SYBR Premix Ex Taq kit (Yeasen) on an AB 7500 Fast Real-Time PCR System (Applied Biosystems). Rat β-actin was used as an internal control. The sequences of primers are listed in Table S1. The fold changes in mRNA expression levels were normalized to β-actin using the ^ΔΔ^Ct method.

### Cytokine analysis by ELISA

Mouse serum was collected from each experimental group. The levels of IL-1β (Invitrogen, 88-7013-88), IL-6 (BD Pharmingen, 554400, 554402) and IL-12 (BD Pharmingen, 551219, 554476) in the serum were quantified by enzyme-linked immunosorbent assay (ELISA) kits according to the manufacturer's protocol.

### Western blot analysis

Rat BMDMs were lysed with RIPA lysis buffer (Beyotime), and protein concentrations were measured using a BCA assay kit (Beyotime). The total proteins were separated by sodium dodecyl sulfate-polyacrylamide gel electrophoresis and transferred to nitrocellulose membranes (Amersham Pharmacia Biotech, Buckinghamshire, UK). After blocking, the membranes were incubated with anti-Bcl-2 (1:1000; Cell Signaling Technology, 3498S), anti-AKT (1:1000; Cell Signaling Technology, 9272S), anti-p-AKT (1:1000; Cell Signaling Technology, 4060S), anti-cleaved PARP (1:1000; Cell Signaling Technology, 9548T), anti-caspase 3 (1:1000; Cell Signaling Technology, 9662S), anti-cleaved caspase 3 (1:1000; Cell Signaling Technology, 9664S), anti-NF-κB p65 (1:1000; Cell Signaling Technology, 8242S), anti-p-NF-κB p65 (1:1000; Cell Signaling Technology, 3033S), anti-ERK (1:1000; Cell Signaling Technology, 4695S), anti-p-ERK (1:1000; Cell Signaling Technology, 4370S), anti-p38 (1:1000; Cell Signaling Technology, 8690S), anti-p-p38 (1:1000; Cell Signaling Technology, 4511S), anti-JNK (1:1000; Abcam, Cambridge, UK, ab179461) or anti-p-JNK (1:1000; Abcam, ab76572). After washing with TBS with Tween 20, horseradish peroxidase (HRP)-conjugated anti-rabbit IgG (1:10,000; Bio-Rad, Richmond, CA, USA, 1706515) was added, and HRP-conjugated monoclonal mouse anti-β-actin (1:10,000; Abcam, ab49900) was used as a control for normalization. Protein signals were detected with an ECL system (Amersham Bioscience, Buckinghamshire, UK) in a ChemiDoc™ MP Imaging System (Bio-Rad).

### Flow cytometry analysis

Surface marker staining was conducted and analyzed using a previously reported method (Zhang et al., 2019b). Briefly, for surface marker, cells were collected and blocked with anti-rat and mouse CD16/CD32 (Fc receptor block; eBioscience, San Diego, CA, USA, 14-0161-86), and stained with fluorescein isothiocyanate (FITC)- and phycoerythrin (PE)-conjugated monoclonal antibodies for membrane molecules. The immunofluorescent antibodies used in this analysis were from BD Biosciences or Thermo Fisher Scientific. Flow cytometry analysis was performed on a CytoFlex analytical cytometer (Beckman Coulter, Brea, CA, USA) or an LSRFortessa analytical cytometer (BD Biosciences), and the data were analyzed with FlowJo software (Tree Star, Ashland, OR, USA).

### Immunofluorescence cytochemistry

Rat NRVMs and BMDMs on coverslips were fixed in 4% PFA for 30 min and permeabilized with 1% Triton X-100 for 10 min. After blocking with 1% bovine serum albumin (BSA) for 1 h, NRVMs were incubated with rabbit anti-Ki67 (1:200; Cell Signaling Technology, 12075S), anti-pH3 (1:200; Cell Signaling Technology, 53348S) and mouse anti-α-actinin (1:200; Cell Signaling Technology, 69758S) at 37°C for 2 h. After washing with 1% PBS-Tween 20, Alexa Fluor 488-conjugated anti-rabbit and Alexa Fluor 555-conjugated anti-mouse secondary antibodies (Abcam, ab150077, ab150118) were added. BMDMs were incubated with rabbit anti-p-NF-κB p65 (1:200; Cell Signaling Technology) at 37°C for 2 h. After washing with 1% PBS-Tween 20, Alexa Fluor 488-conjugated anti-rabbit secondary antibodies were added. Dihydrorhodamine 123 (DHR123; Thermo Fisher Scientific, D23806) staining was applied directly onto the slide for 1 h following the manufacturer's instructions. Negative control reactions were included in each experiment and carried out by replacing primary antibodies with PBS. BMDMs were incubated with rabbit anti-iNOS (Abcam, ab178945), and Alexa Fluor 488-conjugated anti-rabbit secondary antibody was added. Heart tissues were embedded in OCT compound and sectioned at 6 µm on a cryostat. After fixation in PFA, sections were blocked with 1% BSA for 60 min and then incubated with PE-conjugated anti-His36 (1:100; eBioscience, 12-0660-82) at 4°C overnight. All the sections were counterstained with 4′,6-diamidino-2-phenylindole (DAPI; Abcam). Fluorescent cells and tissues were visualized and digital images were captured using an Axio Scan Z1 (Carl Zeiss, Germany).

### LDH release assay

NRVMs were pretreated with H_2_O_2_ (300 μmol/l) in the absence or presence of BCI (1 μmol/l) for 24 h. The supernatants were collected and analyzed for LDH release using an LDH-release kit (Beyotime) following the manufacturer's instructions.

### RNA-seq

RNA-seq of BMDMs was performed by Novogene. Briefly, total RNAs were extracted from BMDMs (*n*=2 or 3 in each group), and sequencing libraries were constructed and carried out on an Illumina NovaSeq 6000. Differential gene expression of control and BCI-treated groups was analyzed using the DESeq2 R package (1.20.0). DESeq2 provides statistical routines for determining digital differential gene expression data using a model based on the negative binomial distribution. The resulting *P*-values were adjusted using the Benjamini and Hochberg approach for controlling the false discovery rate. An adjusted *P*-value of ≤0.05 and |log2(foldchange)|≥1 were set as the threshold for significantly differential gene expression. GO enrichment analysis of differentially expressed genes was implemented by the cluster Profiler R package (3.8.1), in which gene length bias was corrected. GO terms with corrected *P*-values ≤0.05 were considered significantly enriched in differentially expressed genes. The Kyoto Encyclopedia of Genes and Genomes (KEGG) is a database resource for understanding high-level functions and utilities of biological systems, such as the cell, the organism and the ecosystem, from molecule-level information, especially large-scale molecular datasets generated by genome sequencing and other high-throughput experimental technologies (http://www.genome.jp/kegg/). We used the cluster Profiler R package (3.8.1) to test the statistical enrichment of differentially expression genes in KEGG pathways.

### Preparation of PLGA microspheres

The PLGA microspheres were prepared according to a method as previously described (Jaidev et al., 2015). Briefly, 10 mg PLGA was dissolved in 1 ml chloroform (Merck, Germany), and the polymer solution was dispersed into 3 ml of 0.5% polyvinyl alcohol (PVA; w/v) and emulsified using a probe sonicator (VCX130, Vibracell, Sonics, Newtown, CT, USA) for 3 min. The organic solvent was removed by centrifugation at 13,800 ***g*** for 30 min, and the resultant microspheres were collected from the pellet. The pellets were dispersed in milli-Q (Millipore, Burlington, MA, USA) water and lyophilized.

### Preparation of BCI-loaded PLGA microspheres

BCI-loaded PLGA microspheres were developed using the water/oil/water emulsion technique. In brief, 10 mg BCI was dissolved in 5 ml ethanol and dispersed into PLGA solution (25 mg/ml chloroform). The organic solution obtained was sonicated for 30 s, and the resultant primary emulsion was then dispersed into 10 ml of 2% (w/v) PVA and sonicated for 3 min. The secondary emulsion was prepared by sonicating for 30 s and dispersed into 100 ml of 0.3% PVA. This secondary emulsion was stirred for 3 h and then subjected to rotary evaporation to remove chloroform followed by centrifugation at 13,800 ***g*** for 30 min. This process was repeated twice to remove PVA. The final pellet was dispersed in milli-Q water and lyophilized to obtain a powder formulation, which was stored in a desiccator until further use.

### Statistical analysis

One-way ANOVA followed by Dunnett's multiple comparison test was used to compare parameters involving multiple groups using Prism 7.0 (GraphPad Software, San Diego, CA, USA) statistical software. *P*<0.05 was considered significant.

## Supplementary Material

10.1242/dmm.049662_sup1 Supplementary information
