# Supplementary material for: A DUSP6 inhibitor suppresses inflammatory cardiac remodeling and improves heart function after myocardial infarction
Source: Dis Model Mech. 2022 Dec 7;16(5):dmm049662. doi: 10.1242/dmm.049662 (PMC9789401; doi:10.1242/dmm.049662)
Supplement: Supplementary information [file dmm-16-049662-s1.pdf]

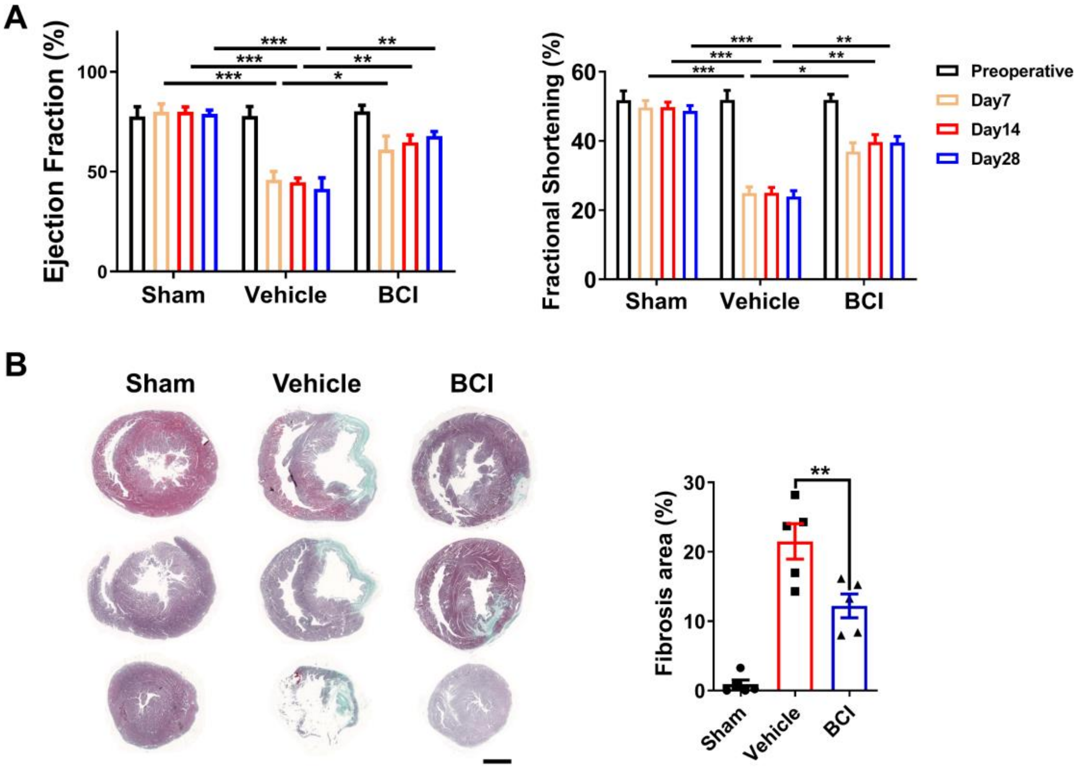

Fig. S1. BCI treatment, immediately after MI, improves heart function and decreased cardiac fibrosis. (A) ECHO data showing ejection fraction and fractional shortening of sham-operated and 4 weeks post-MI rats treated with DMSO or BCI, which DMSO/BCI treatment was carried out immediately after MI (n = 8–9 per group). (B) Masson's trichrome staining and quantification of the Masson's trichrome-stained fibrotic area in sham, vehicle, and BCI-treated hearts at 28 days post-MI (Scale bar, 3 mm; n = 5 per group). Mean ± s.e.m.; ns, not significant; *P <0.05; **P <0.01, ***P <0.001.

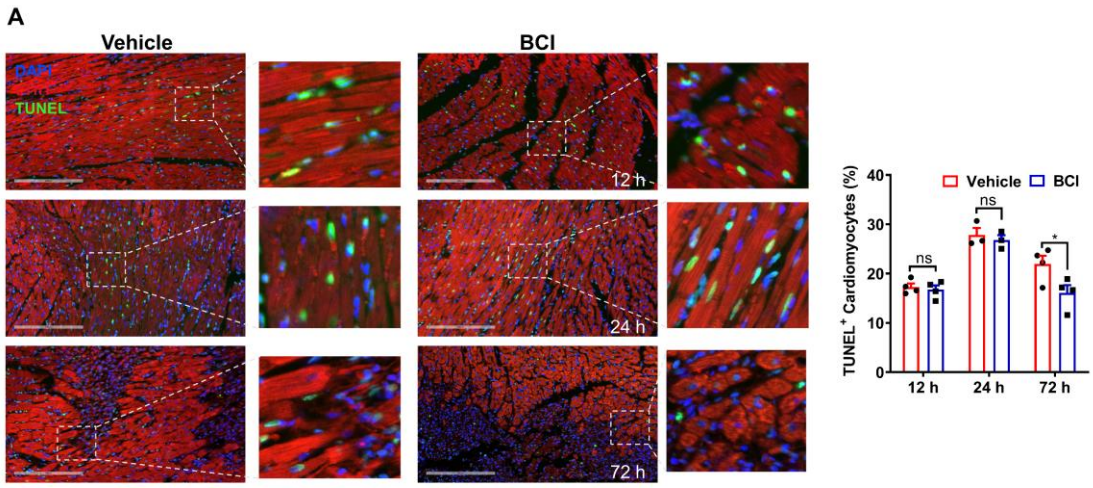

Fig. S2. BCI inhibits cardiomyocyte apoptosis at 72 h post MI, but not at 12 h and 24 h post MI. (A) Immunostaining and percentage of TUNEL-positive CMs in the infarcted zone of sham-operated, vehicle, and BCI-treated heart sections at 12 h, 24 h, and 72 h post-MI. Scale bars, 200 µm. (n = 3–4 per group). Mean ± s.e.m.; ns, not significant; *P <0.05.

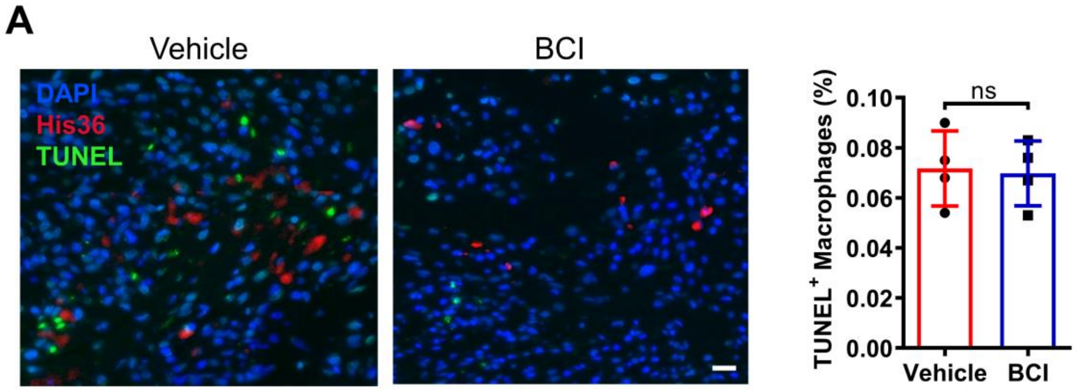

Fig. S3. BCI treatment has no effect on macrophage death of infarcted rat hearts at 7 days post MI. (A) Immunostaining and quantification of TUNEL-positive macrophages in the infarcted zone of vehicle and BCI-treated heart sections (Scale bar, 50 μm; n = 4 per group). ns, not significant.

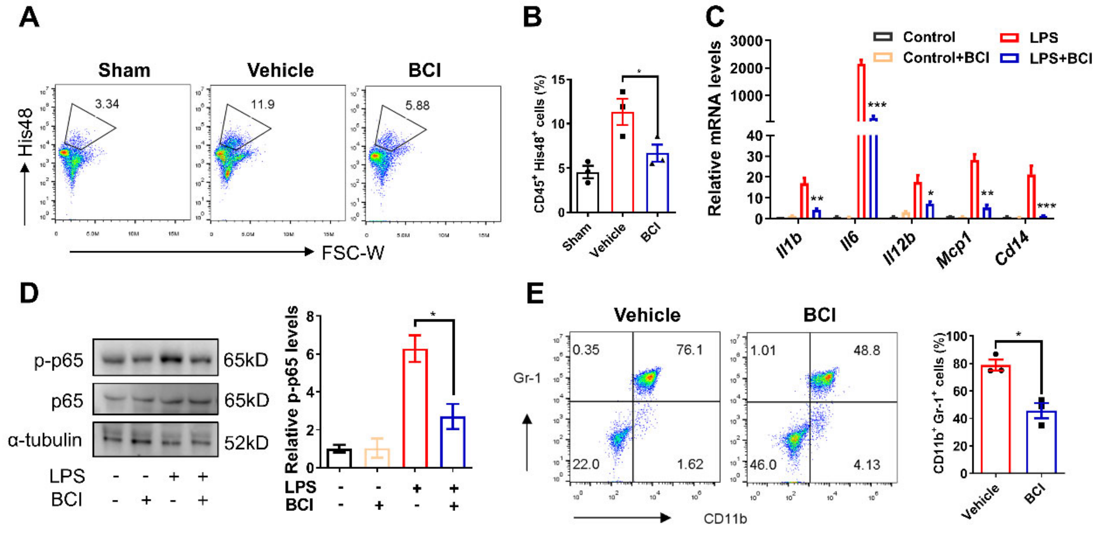

Fig. S4. BCI treatment inhibits neutrophil recruitment in infarcted rat hearts and LPS-induced mouse abdominal cells. (A, B) Percentages of His48⁺ neutrophils in the LV of rats at 7 days post-MI by flow cytometry (A), and statistics of His48⁺ neutrophils (B) (n = 3 per group and 2 hearts each group). (C) qRT-PCR showing expression levels of LPS-induced *Il1b*, *Il6*, *Il12b*, *Mcp1* and *Cd14* mRNAs in ABNs treated with DMSO or BCI (n = 3 per group). (D) Western blots and quantification of LPS-induced p-p65 and p65 in ABNs with DMSO or BCI (n = 3 per group). (E) Percentages of LPS-induced CD11b⁺ Gr-1⁺ neutrophils in mouse abdominal cells by flow cytometry, and statistics of CD11b⁺ Gr-1⁺ neutrophils (n = 3 per group and combined abdominal cells of 2 mice per sample). One-way ANOVA followed by Dunnett's multiple comparison test; mean± s.e.m.; *P <0.05; **P <0.01, ***P <0.001.

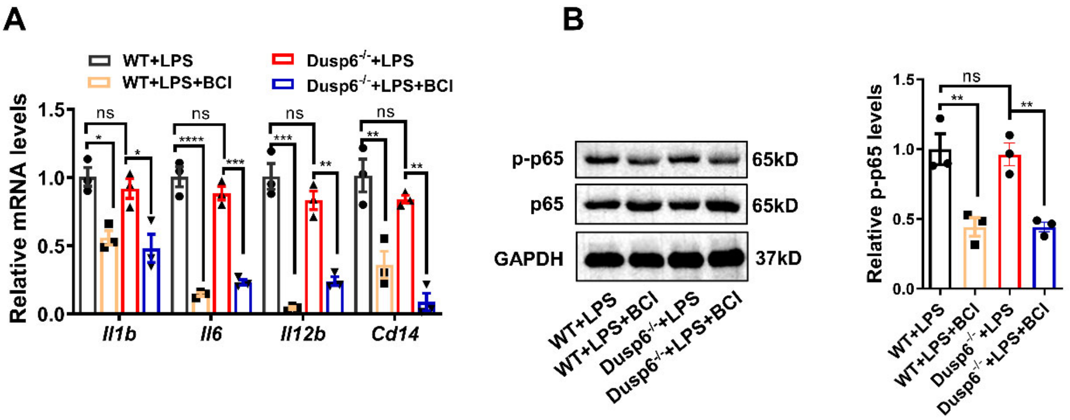

Fig. S5. BCI inhibits macrophage inflammation independent of DUSP6. (A) qRT- PCR showing expression levels of LPS-induced *Il1b*, *Il6*, *Il12b* and *Cd14* mRNAs in WT or *Dusp6*$^{-/-}$ BMDMs with DMSO or BCI (n = 3 per group). (B) Western blots and quantification of LPS-induced p-p65 and p65 in WT or *Dusp6*$^{-/-}$ BMDMs with DMSO or BCI (n = 3 per group). Mean ± s.e.m.; *P <0.05; **P <0.01, ***P <0.001, ****P <0.0001.

Disease Models & Mechanisms • Supplementary information

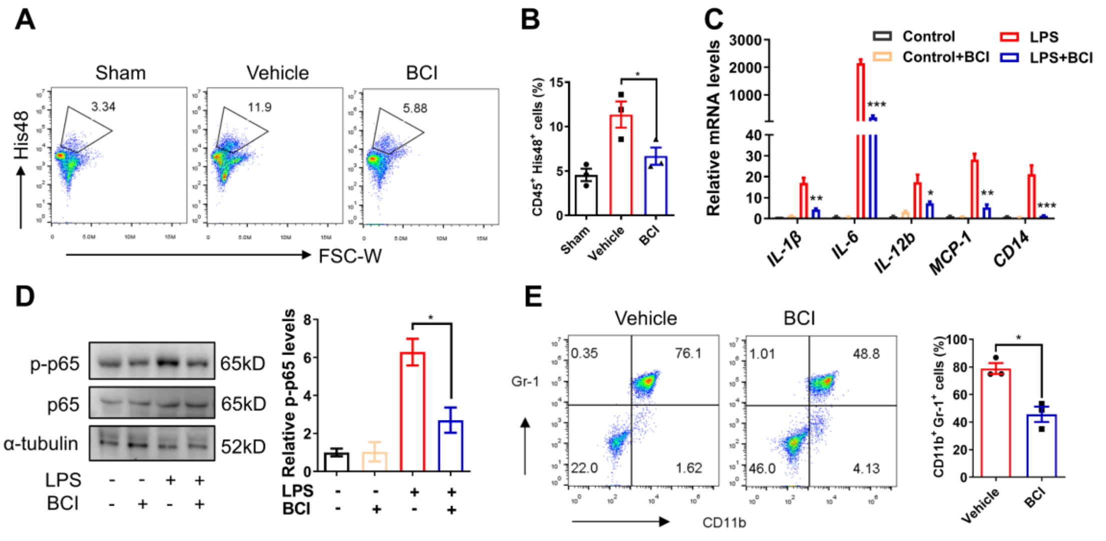

Fig. S4. BCI treatment inhibits neutrophil recruitment in infarcted rat hearts and LPS-induced mouse abdominal cells. (A, B) Percentages of His48+ neutrophils in the LV of rats at 7 days post-MI by flow cytometry (A), and statistics of His48^{+} neutrophils (B) (n = 3 per group and 2 hearts each group). (C) qRT-PCR showing expression levels of LPS-induced *IL-1β*, *IL-6*, *IL-12b*, *Mcp1* and *CD14* mRNAs in ABNs treated with DMSO or BCI (n = 3 per group). (D) Western blots and quantification of LPS-induced p-p65 and p65 in ABNs with DMSO or BCI (n = 3 per group). (E) Percentages of LPS-induced CD11b^{+} Gr-1^{+} neutrophils in mouse abdominal cells by flow cytometry (G), and statistics of CD11b^{+} Gr-1^{+} neutrophils (H) (n = 3 per group and combined abdominal cells of 2 mice per sample). One-way ANOVA followed by Dunnett's multiple comparison test; Mean ± s.e.m.; *P <0.05; **P <0.01, ***P <0.001.

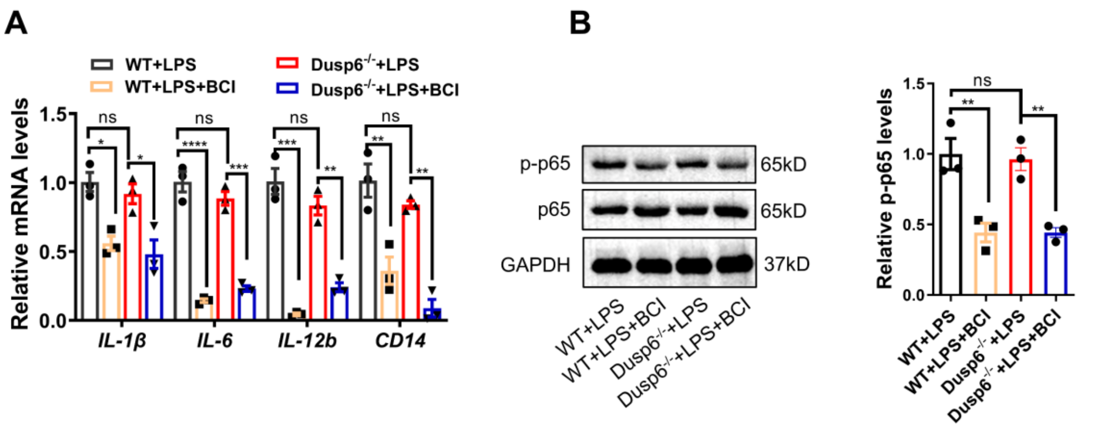

Fig. S5. BCI inhibits macrophage inflammation independent of DUSP6. (A) qRT-PCR showing expression levels of LPS-induced *IL-1β*, *IL-6*, *IL-12b* and *CD14* mRNAs in WT or *Dusp6*$^{-/-}$ BMDMs with DMSO or BCI (n = 3 per group). (B) Western blots and quantification of LPS-induced p-p65 and p65 in WT or *Dusp6*$^{-/-}$ BMDMs with DMSO or BCI (n = 3 per group). Mean ± s.e.m.; *P <0.05; **P <0.01, ***P <0.001.

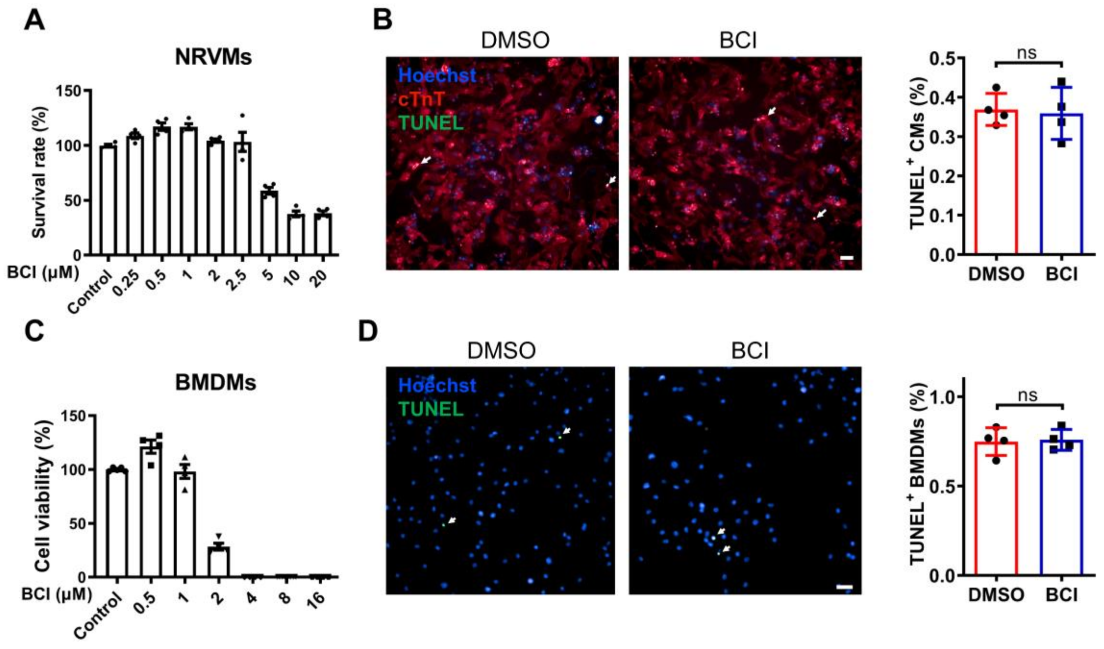

Fig. S6. Dose-dependent toxicity of BCI to NRVMs and BMDMs. (A) Cytotoxicity of different concentrations of BCI on NRVMs (n = 4 per group). (B) Immunostaining and statistics showing TUNEL-positive NRVMs after DMSO or 1 μmol L^{-1} BCI treatment. Scale bars, 50 μm. (n = 4 per group). (C) Cytotoxicity of different concentrations of BCI on BMDMs (n = 4 per group). (D) Immunostaining and statistics showing TUNEL-positive BMDMs after DMSO or 1 μmol L-1 BCI-treated. Scale bars, 50 μm. (n = 4 per group). ns, not significant.

Disease Models & Mechanisms • Supplementary information

Table S1. Primers used in real-time PCR analysis

Gene	Primer	Sequence (5'-3')
Rat β-actin	Forward	AACCTTCTTGCAGCTCCTCC
	Reverse	TACCCACCATCACACCCTGG
Rat Inos	Forward	TGAAGCACTTTGGGTGACCA
	Reverse	TATACACGGAAGGGCCAAGC
Rat IL-1β	Reverse	GACTTCACCATGGAACCCGT
	Forward	GGAGACTGCCCATTCTCGAC
Rat IL-6	Forward	ACAAGTCCGGAGAGGAGACT
	Reverse	TTGCCATTGCACAACTCTTTTC
Rat IL-12b	Forward	ATCATCAAACCGGACCCACC
	Reverse	CAGGAGTCAGGGTACTCCCA
Rat Cd14	Forward	TCAGAATCTACCGACCATGAAGC
	Reverse	GCTCCAGCCCAGTGAAAGAT
Rat Mcp-1	Forward	GATCCCAATGAGTCGGCTGG
	Reverse	ACAGAAGTGCTTGAGGTGGTT
Rat Ccr2	Forward	CAACCTGGCCATCTCTGACC
	Reverse	AAGTGCATGTCAACCACACAG
Rat Ccl4	Forward	GCTGTCAGCACCAATAGGCT
	Reverse	AGTTCCGATGAATCTTCCGGG
Rat Cxcl9	Forward	GACTCCAGCACGGTGACTTA
	Reverse	ATGCAGGAGCATCGCTGATT
Rat Mnda	Forward	GGTGGGGAGTGGAAAATGGT
	Reverse	CGAGCTCTGGTGACCTTGAT
Rat Egr1	Forward	AACAACCCTACGAGCACCTG
	Reverse	AAAGGGGTTCAGGCCACAAA
Rat Grb2	Forward	TTACGGAATCTCGCCGCTAC
	Reverse	AAAGGCTTCATGGGATGGGG
Rat Retnla	Forward	CTGGCAAGGTCCTGGAAACT
	Reverse	GCATAGGCCCAGTCAACGAT
Rat Arg1	Forward	GTGCCCTCTGTCTTTTAGGG
	Reverse	CAGACCGTGGGTTCTTCACA
Rat Ym1	Forward	ATGAGATCCCCCAGCTGTCT
	Reverse	GGTTACGGTCACATGGGTGT

Disease Models & Mechanisms • Supplementary information
